# Unplanned Perioperative Reoperation Following Pulmonary Resection in Lung Cancer Patients: A Report of a Single‐Center Experience

**DOI:** 10.1111/crj.13810

**Published:** 2024-08-06

**Authors:** Hongxiang Feng, Yue Zhao, Chaoyang Liang, Yuhui Shi, Deruo Liu, Jin Zhang, Zhenrong Zhang

**Affiliations:** ^1^ Department of Thoracic Surgery China‐Japan Friendship Hospital Beijing China

**Keywords:** lung cancer, perioperative reoperation, pulmonary resection, secondary surgery

## Abstract

**Background:**

Pulmonary resection is an important part of comprehensive treatment of lung cancer. Despite the progress in recent thoracic surgery, reoperation is occasionally inevitable for managing severe perioperative complications. This study aimed to investigate the incidence and causes of perioperative reoperation in lung cancer patients.

**Methods:**

We retrospectively collected patients who underwent reoperation following pulmonary resection from January 2010 to February 2021 in China‐Japan Friendship Hospital.

**Results:**

Among the 5032 lung cancer patients who received primary pulmonary resection in our institute, 37 patients underwent perioperative reoperation with the rate being 0.74%. Lobectomy was the most frequently executed procedure (56.8%). The mean duration of the primary surgery was 143.6 ± 65.1 min. About half of the cases received secondary surgery within 24 h of the primary surgery, whereas only one case underwent secondary surgery 30 days after the primary surgery (due to chylous leakage). The major causes of the reoperation were bleeding (73.0%), chylous leakage (13.5%), lobar torsion (5.4%), air leakage (2.7%), atelectasis (2.9%), and cardiac herniation (2.7%).

**Conclusion:**

The most prevalent reasons for unplanned reoperation following pulmonary resection in lung cancer patients include bleeding, chylous leakage, and lobar torsion. The strict control of the surgical indications and standardization of surgical procedures are fundamental to reduce unplanned secondary operations after pulmonary resections. Timely identification of the need to secondary surgery is also important to ensure patients' safety.

## Graphical abstract

The most prevalent reasons for unplanned reoperation following pulmonary resection in lung cancer patients include bleeding, chylous leakage, and lobar torsion. Timely identification of the need to secondary surgery is also important to ensure patients' safety.

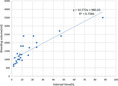

## 1 Introduction

Lung cancer is one of the leading causes of cancer‐related mortality around the world. Although new techniques such as molecular targeted therapy and immune therapy are progressing, arousing more attention of scientists and clinicians, surgical operation remains the gold standard for treating early‐stage lung cancer. In the case of unresectable tumors, surgery is sometimes performed to get enough biopsy sample or to reduce tumor burden. Surgical procedures of pulmonary resection are diverse, including pulmonary wedge resection, segmental resection, lobectomy, and pneumonectomy. Mediastinal lymph node dissection is also common in surgery for lung cancer patients. In addition to traditional open surgery, nowadays, minimal invasion techniques such as thoracoscopy plays an increasingly important role in lung cancer treatment [1]. However, some patients may undergo unplanned reoperation after lung cancer surgery due to the direct or indirect complications of the original surgery. Investigating possible contributors of unplanned perioperative reoperation may help to reduce the incidence of secondary operation and thus avoid unnecessary patient injury. Therefore, in this study, after reviewing 5032 cases of pulmonary resection that we performed in lung cancer patients in our center, we summarized the incidence and analyzed the causes of perioperative reoperation. We present the following article in accordance with the STROBE reporting checklist.

### 1.1 Methods

All 5032 lung cancer patients undergoing pulmonary resection in our department between January 2010 and February 2021 were enrolled in this study. All operation procedures were determined after discussions among participating surgeons based on the preoperative assessments of every patient and were performed under general anesthesia. Surgical methods include pulmonary wedge resection, segmental resection, lobectomy, and pneumonectomy. Most patients underwent video‐assisted thoracic surgery when feasible. This study was approved by the Ethics Committee of China‐Japan Friendship Hospital (No. 2021‐140‐K98), and individual consent for this retrospective analysis was waived.

All patients with lung cancer who underwent perioperative reoperation with the primary surgery were included in the study. Data of all lung cancer patients who underwent perioperative reoperations after pulmonary resection for the management of complications were collected retrospectively from the database of our department. The medical histories of all patients were reviewed. Patient characteristics include age, gender, smoking history, and major comorbidity. Tumor characteristics include tumor site, pathological type, and TNM stage. Details regarding surgery and subsequent treatment were reviewed carefully, including surgical method, duration of surgery, intraoperative bleeding, major postoperative complication, and the interval time between the primary and secondary surgery. In our study, the perioperative reoperation was defined as a secondary operation to manage severe complications within 30 days of the primary surgery. For patients with suspective bleeding, intensive care would be applied, and patients' vital signs and pleural drainage would be closely monitored. Reoperation happens when hemorrhagic drainage was more than 200 mL for 3 consecutive hours. For patients with chylous leakage or air leakage, reoperation takes place when conservative treatments were not effective for 2 weeks, whereas for patients with lobar torsion or cardiac herniation, operation is performed as soon as the diagnosis is confirmed. Planned reoperations at the time of the primary surgery were excluded. Besides, extended re‐resection due to the discrepancy between the intraoperative frozen section and the postoperative pathological examination and minor complications that could be dealt with through percutaneous or endoscopic interventions such as chest puncture and bronchoscopic sputum aspiration were also excluded in this study. Thus, unplanned reoperation underlined the severity and urgency of the postoperative complication that needs to be addressed by surgical procedures.

### 1.2 Statistical Analysis

IBM SPSS Statistics for Windows, Version 26.0 (IBM Corp., Armonk, NY, USA), was used for statistical analysis. Continuous variables were presented as mean ± standard deviation and categorical variables as the number of cases and percentage. Comparison of reoperation rates between open surgery and minimally invasive surgery was performed using chi square test. All tests were two‐tailed, and *p* < 0.05 was considered statistically significant.

## 2 Results

### 2.1 Patient Characteristics

From January 2010 to February 2021, 5032 pulmonary resections were performed in our institution, among which 37 cases underwent perioperative reoperation. Demographics and the baseline characteristics of 37 patients are presented in Table 1. The mean age was 59.4 ± 7.5 years (range, 43–76 years). There were 28 (75.7%) males and 9 (24.3%) females. About half of the patients (45.9%) had at least one comorbidity, and the most common comorbidity was hypertension. Five patients have multiple tumor nodules distributed in different pulmonary lobes, and they received bilobectomy or wedge resection together with pneumonectomy. Adenocarcinoma was the most prevalent pathological type, and 43.2% cases were invasive or minimally invasive subtypes under postoperative pathological examination.

**TABLE 1:** Clinical and surgical characteristics of 37 patients.

Characteristics	Data
Age, y	59.4 ± 7.5
Male, %	28 (75.7)
Smoking history, %	18 (48.6)
Comorbidities, %	
Hypertension	11 (29.8)
Diabetes	3 (8.1)
Coronary heart disease	3 (8.1)
Others	5 (13.5)
Tumor location, %	
Upper lobe	16 (43.2)
Middle lobe	2 (5.4)
Lower lobe	13 (35.1)
Multiple tumor nodules	5 (13.5)
Hilus pulmonis	1 (2.7)
Tumor side, %	
Left	7 (18.9)
Right	29 (78.4)
Bilateral	1 (2.7)
Tumor pathology, %	
Adenocarcinoma	24 (64.9)
Squamous cell carcinomas	10 (27.0)
Small cell lung cancer	2 (5.4)
Synovial sarcoma	1 (2.7)
TNM staging, %	
I	25 (67.6)
II	3 (8.1)
III	7 (18.9)
IV	2 (5.4)
Type of operative procedures, %	
Wedge resection	7 (18.9)
Anatomical pulmonary segmentectomy	2 (5.4)
Lobe resection	21 (56.8)
Sleeve lobectomy	3 (8.1)
Bilobectomy	3 (8.1)
Pneumonectomy	1 (2.7)
Mediastinal lymph node dissection, %	14 (37.8)
Open surgery, %	16 (43.2)
Duration of primary surgery (min)	143.6 ± 65.1
Interval between primary and secondary surgery (d)	
< 1	19 (51.4)
1–3	7 (18.9)
3–7	5 (13.5)
7–21	5 (13.5)
21–30	1 (2.7)

### 2.2 Primary Surgery

**FIGURE 1:**
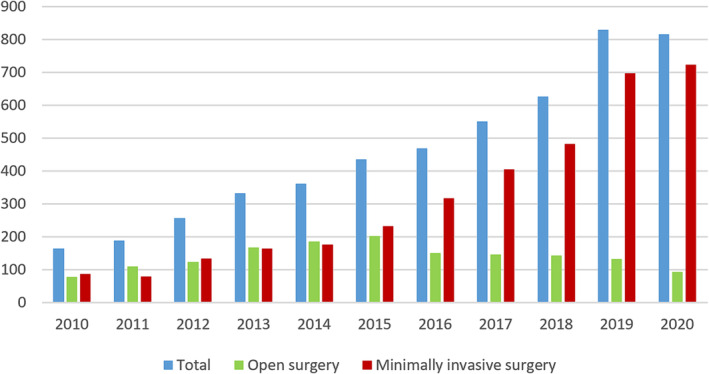
Number of open surgery and minimally invasive surgery from 2010 to 2020.

In the 37 cases, the surgical methods such as wedge resection, anatomical pulmonary segmentectomy, lobe resection, sleeve lobectomy, bilobectomy, or pneumonectomy were performed according to the preoperative assessments of each patient after careful discussion by all participating surgeons. Lobectomy was the most frequently executed operation procedure (56.8%). Of the 37 patients, 16 patients received open surgery, and the rest 21 cases underwent minimally invasive thoracoscopic surgery. Fourteen patients (37.8%) received mediastinal lymph node dissection. The duration time of the primary surgery was 143.6 ± 65.1 min. Details of the surgical characteristics were shown in Table 1. During the 10‐year period, the number of minimally invasive surgeries has gradually increased, whereas open surgeries have gradually decreased, and the percentage of open surgery has decreased from about 50% in 2010 to about 10% in 2020 (Figure 1). Furthermore, no significant statistical difference was observed in terms of the reoperation rate between open surgery and minimally invasive surgery (0.66% vs. 0.91%, *p* = 0.328).

### 2.3 Unplanned Reoperations

About half of the cases (19 cases, 51.4%) received secondary surgery within 24 h of the primary surgery, whereas only one case underwent secondary surgery 30 days after the primary surgery (due to chylous leakage). The major causes of the reoperation were bleeding (27 cases, 73.0%), chylous leakage (5 cases, 13.5%), lobar torsion (2 cases, 5.4%), air leakage (1 case, 2.7%), atelectasis (1 case, 2.7%), and cardiac herniation (1 case, 2.7%). Postoperative bleeding was the most frequent surgical complication leading to an unplanned secondary surgery (27 cases, 73%). The most common seen bleeding area included trocar (29.6%), lymph node area (22.2%), and pleura (5%).

**FIGURE 2:**
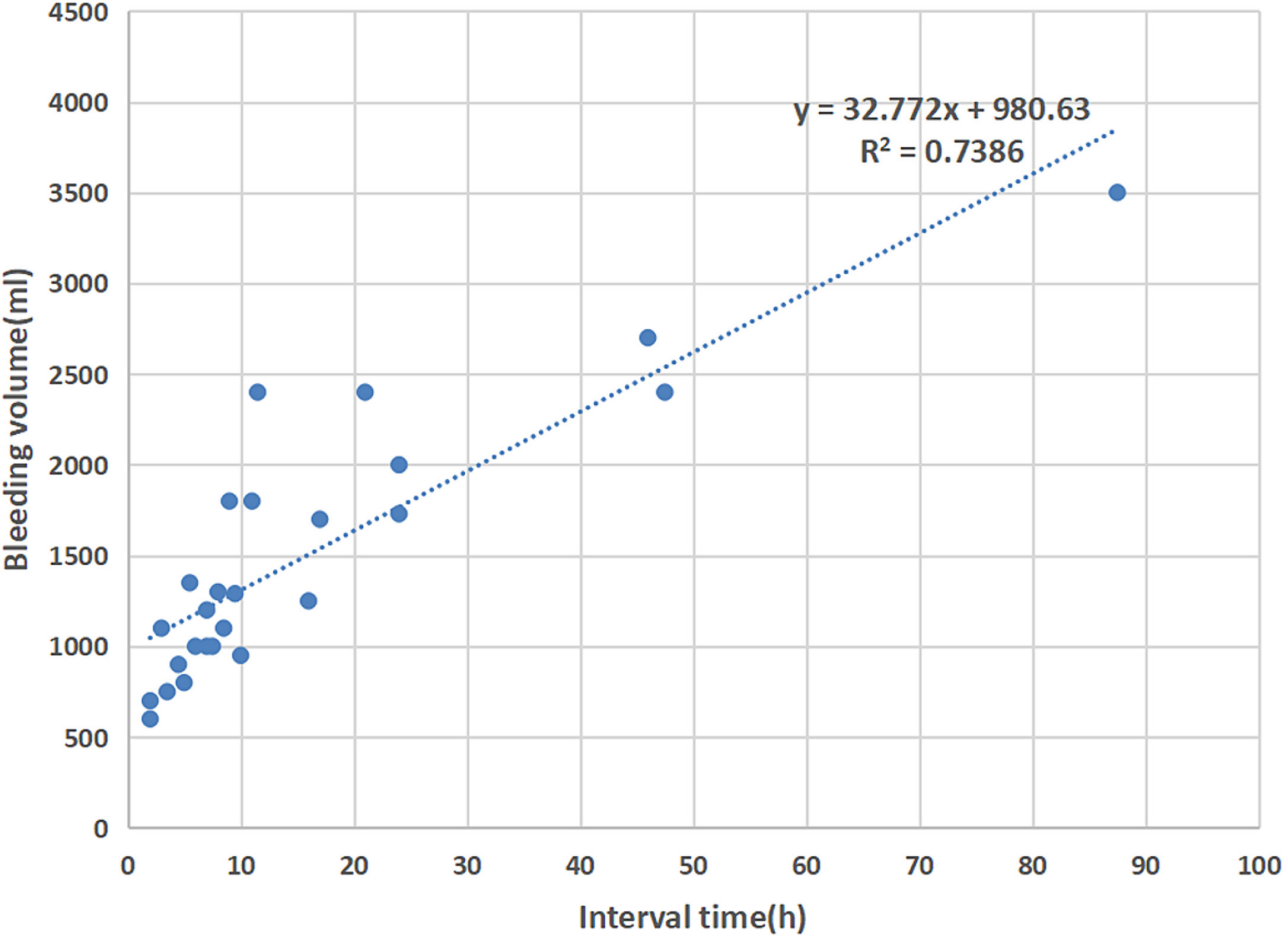
Scatter diagram showing the relationship between the time interval and bleeding volume.

In our experience, most of the severe postoperative bleeding was within 72 h after the primary surgery (26 cases, 70.3%). There was only one case that received a second operation due to delayed hemorrhage 21 days after the primary surgery. In the 26 patients underwent secondary operation within 72 h after the primary surgery, we estimated the bleeding volume throughout the interval of the two surgeries. The bleeding volume correlates positively with the time interval (Figure 2). Notably, the actual blood loss could be underestimated due to blood clots in the thoracic cavity and obstruction of the drainage tube, especially in those cases where the “wait‐and‐see” strategy was applied.

**FIGURE 3:**
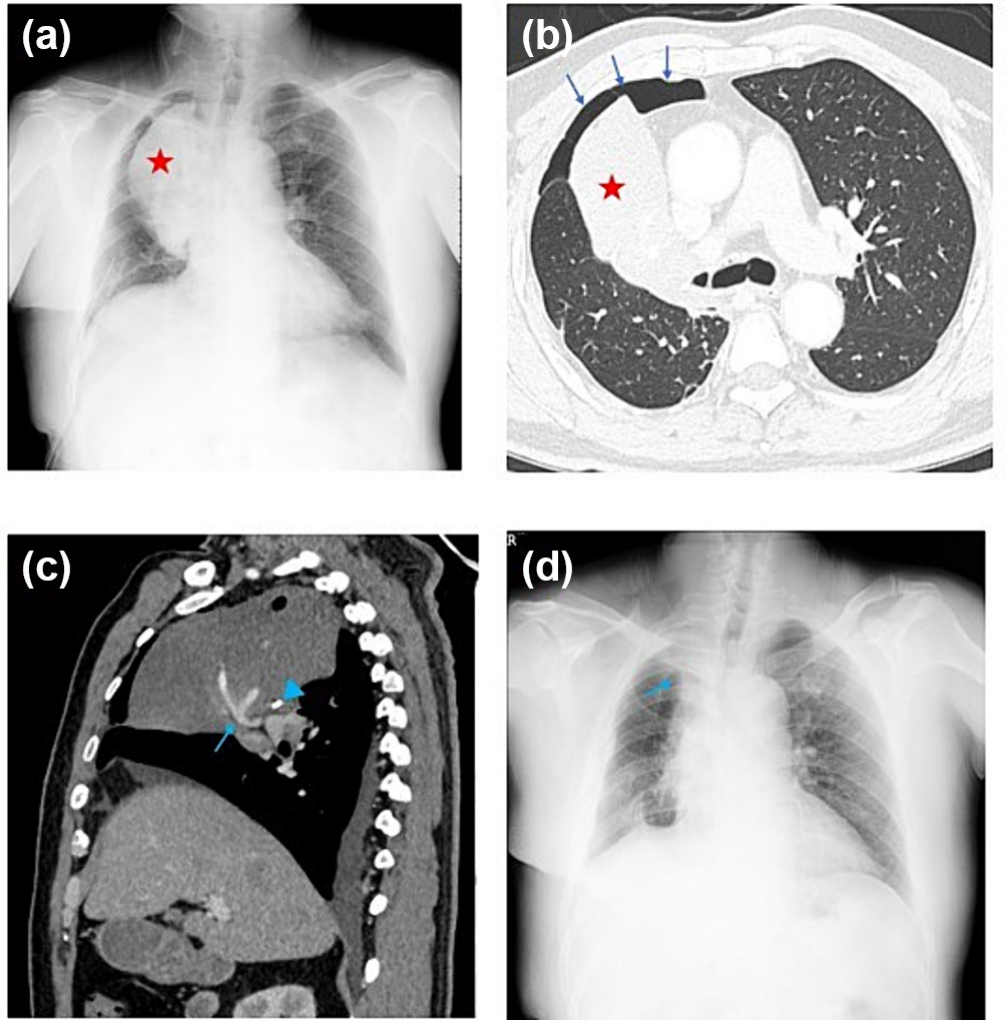
Right middle lobe torsion in a 61‐year‐old man who had undergone right upper lobectomy for invasive adenocarcinoma. (a) Chest radiograph after right upper lobectomy shows right perihilar opacification (red star) with well‐defined margin, a right chest drain, and a right central catheter. (b) Axial image of the postoperative contrast‐enhanced computed tomography (CT) scan on lung window. The middle lobe tapes towards the hilum with complete consolidation (red star). Note also the postoperative pneumothorax (blue arrows). (c) Sagittal reformat of postoperative contrast‐enhanced CT scan on soft tissue window. A tortured pulmonary artery (blue arrow) and hilar surgical sutures (blue arrowhead) can be observed. (d) Chest radiograph after the secondary operation shows increased density (blue arrow) adjacent to the right hilum but no lobar parenchymal consolidation.

Another uncommon but severe complication after pulmonary resection is lobar torsion. In our study, there were two cases of lobar torsion in need of a secondary operation. In both cases, the patients received pulmonary resection of the right upper lobe, and the torsion of the right middle lobe was identified during the secondary operation. One typical case was a 61‐year‐old male. He underwent thoracoscopic lobectomy of the right upper lobe and mediastinal lymph node dissection during the primary surgery. The patient complained of difficulty in expectorating on the first postoperative day, and the bronchoscopic examination has showed stenosis of the right middle lobe bronchus. Six days after the primary surgery, the patient presented with hemoptysis. Chest x‐ray indicated right perihilar opacification with a well‐defined margin (Figure 3a). The symptoms did not relieve 12 days after the primary surgery, and the computed tomography (CT) scan showed complete consolidation of the right middle lobe and a tortured pulmonary artery can be observed (Figure 3b,c). The patient received a lobectomy of the right middle lobe 13 days after the primary surgery, and the torsion of the right middle lobe was identified. He recovered well after the secondary surgery and was discharged from the hospital 6 days later. Chest x‐ray showed increased density adjacent to the right hilum but no lobar parenchymal consolidation (Figure 3d).

After reoperation for different reasons, the patients' vital signs and other parameters were closely monitored, and all patients made a fully recovery and got discharged. No perioperative death occurred. Their length of stay (LOS) after reoperation were displayed in Table 2. From the table, we can see that unplanned return to the operation room (UROR) would prolong patients' LOS. Patients who were reoperated for air leakage had the longest LOS, and patients who were reoperated for bleeding, which is the most common reason, had an average 4.7 days of LOS longer. Other patients reoperated for lobar torsion, chylous leakage, atelectasis, and cardiac herniation had an average longer LOS of 5.5, 5.8, 4, and 3 days, respectively.

**TABLE 2:** Patients' length of stay after reoperation.

Reasons for reoperation	Bleeding	Lobar torsion	Chylous leakage	Air leakage	Atelectasis	Cardiac herniation
Average length of stay after reoperation (d)	4.7	5.5	5.8	7	4	3

## 3 Discussion

Thoracic surgery is a common and effective therapeutic procedure for lung cancer patients. Minimally invasive approaches such as video‐assisted thoracoscopic surgery (VATS) have improved the safety and feasibility of lung cancer surgery, with less surgical trauma and equivalent or even better prognosis [2]. However, postoperative complications need to be reassessed in modern thoracic surgery. Most of the postoperative complications could be managed conservatively or with percutaneous/endoscopic interventions, whereas in patients with severe or life‐threatening complications, reoperation is a remedial approach to facilitate recovery.

UROR is defined as the reoperation due to a complication or an untoward outcome related to the initial surgery and is used as an indicator of surgical care quality. Another single center, retrospective, observational study was performed in the Department of Thoracic Surgery, IRCCS European Institute of Oncology, Italy. The UROR rate was 1.76% (71 cases out of 4012). The indications for reoperation were hemothorax (54.9%), bronchial fistula (26.8%), empyema (4.2%), prolonged air leaks (4.2%), wound dehiscence (2.9%), and other indications (7%) [3]. Here, we reviewed surgical data of lung cancer patients in our department to investigate the potential causes of unplanned reoperation after pulmonary resection. The major causes of the reoperation were bleeding (73.0%), chylous leakage (13.5%), lobar torsion (5.4%), air leakage (2.7%), atelectasis (2.9%), and cardiac herniation (2.7%). Similar to the study mentioned above, bleeding was the leading cause of reoperation. Possible explanation could be that blood vessels in patients with tumors are often more brittle. In addition, surgeons may pay more attention to the thoroughness of tumor resection and lymph node dissection when performing oncologic thoracic surgery. Both preventions of severe complications in the primary surgery and timely identification of the need for a secondary surgery are important to ensure patients' safety.

In our study, the leading cause of unplanned reoperation after pulmonary resection is postoperative bleeding, which was the same as the previous study [4]. Postoperative bleeding is an uncommon early complication with an incidence of merely 0.1%–0.3% [4, 5]. Early diagnosis and precise assessment of the hemothorax are crucial in the management of postoperative bleeding. We found that most of the postoperative bleedings were within 24 h after the primary surgery. Thus, it is important to intensively monitor the vital sign, drainage volume, and routine blood tests, especially in the first few days after operation. Most of the postoperative bleeding was due to technical problems related to the primary surgery. It is always important to thoroughly inspect before closing the chest. Chylothorax is a potentially severe complication after lung resection. The incidence of chylothorax after pulmonary resection is reported to range from 0.3% to 2.3% [6, 7]. Milky white chest drainage may be the first sign of chylous leakage. Further confirmation could be the presence of triglycerides (> 110 mg/dL) in the pleural fluid [8]. Most patients with chylothorax could be treated conservatively by total parenteral nutrition and fluid drainage. In our experience, chylous leakage was most commonly seen at 1–3 days postoperation, although the surgical intervention was usually performed at 7–21 days after the primary surgery, therefore leaving time windows for conservative treatments. Persistent air leak is the most common complication following all types of lung resection and may lead to prolonged intubation of the chest drainage and hospital stay. The rate of persistent air leak is reported to be 15%–18% [9, 10]. Wedge resection is considered to be a risk factor for air leak because of the high pressure on the staple line [11]. Reoperation is only considered when other approaches failed. Lobar torsion is a rare but life‐threatening complication with an incidence of 0.09%–0.3% [12]. Lobar torsion may involve the twist of arteries, veins, lymphatic tubes, and airways. It is suggested to perform the resection without detorsion to prevent inflammatory factors that build up from the irreversible ischemic damage during the torsion from leaking into the rest of the body [13]. Delayed treatment may lead to complications such as thrombus, pneumonia, air leaks, or emphysema. Serial radiological imaging is critical in identifying lobar torsion, and chest CT is a better way to diagnose lobar torsion. In our study, the two cases of lobar torsion were both related to the right middle lobe post right upper lobectomy. Unfortunately, the torsion of the pulmonary lobe was not recognized in time due to the unspecific symptoms. The affected lobe was found to have irreversible necrosis during the secondary operation, and both cases had to receive lobectomy of the middle lobe. In our experience, lung tissue stapling or lobe pneumopexy should be considered to avoid pulmonary torsion during the primary surgery.

UROR will prolong patients' LOS and increase their medical expenses according to our data. Otherwise, patients who underwent UROR were reported to have a higher mortality, and the most lethal complication was bronchopleural fistula [3]. However, in our study, patients who underwent UROR all fully recovered with no perioperative death occurred, and no bronchopleural fistula‐related UROR was observed. For this reason, we thought that this might contribute to timely identification of acute postoperative bleeding and proper decision on “wait‐and‐see” or quick reoperation to save lives. For the bronchopleural fistula, surgeries for early‐stage lung cancer and small pulmonary nodules grow rapidly nowadays, so minimally invasive video‐assisted thoracic surgeries with advanced surgical instruments are increasing too, which contributed a lot to the prevention of postoperative bronchopleural fistula. Besides, bronchopleural fistula mainly happens after sleeve lobectomy or sleeve bilobectomy in our institution when bronchial anastomoses are required. The increase of minimally invasive surgeries and the decrease of sleeve lobectomies together lower the rate of bronchopleural fistula. Even though a few bronchial fistulas happened during the past decade, considering the acute inflammatory of the original anastomoses, a second surgery would not be chosen. Instead, relying on the support from the pulmonary and critical care medicine, a bronchial stent would be placed for conservative therapy, and the final effect was pretty good. That is why no UROR for bronchial fistula was observed in our cohort.

## 4 Conclusions

In conclusion, the incidence of unplanned reoperation after pulmonary resection is low in our institute. The most common reasons for unplanned reoperation following pulmonary resection in lung cancer patients include bleeding, chylous leakage, lobar torsion, and air leakage. First of all, improving surgical techniques and double‐checking before ending the surgery are fundamental to reduce the incidence of postoperative complications. On top of that, early and precise identification of the severe complications may improve surgical outcomes.

## Author Contributions

All authors contributed to the study conception and design. F.H.X. and Z.Y. performed material preparation and data collection. F.H.X. and L.C.Y. prepared the figures and tables. S.Y.H. followed up the patients. F.H.X. and Z.Y. wrote the manuscript. Z.Y., L.D.R., and Z.J. revised the manuscript. All authors read and approved the final manuscript.

## Ethics Statement

The study was approved by the Institutional Review Board of the China‐Japan Friendship Hospital (No. 2021‐140‐K98). All methods were carried out in accordance with relevant guidelines and regulations. Consent forms for enrolled patients were waived for a retrospective and non‐interventional study.

## Conflicts of Interest

The authors declare no conflicts of interest.

## Data Availability

The datasets used and/or analyzed during the current study are available from the corresponding author on reasonable request.
